# Longitudinal assessment of amyloid-beta deposition in initially amyloid-negative non-demented individuals with [11C]-PIB PET imaging

**DOI:** 10.1097/MD.0000000000027055

**Published:** 2021-09-03

**Authors:** Shizuo Hatashita, Daichi Wakebe

**Affiliations:** Shonan-Atsugi Hospital, Atsugi, Japan.

**Keywords:** [11C]-PIB, Alzheimer's disease, amyloid beta, amyloid-negative, PET

## Abstract

This study aimed to assess the longitudinal changes in amyloid beta (Aβ) deposition in cortical regions with [11C]-PIB PET in initially amyloid-negative non-demented subjects and evaluate whether amyloid-negative subjects convert to amyloid-positive.

Sixteen cognitively normal (CN) and 7 mild cognitive impairment (MCI) subjects (aged 60–75 years), who were amyloid-negative at baseline, underwent 60-minute dynamic [11C]-PIB PET and cognitive assessment over 5.0 to 9.4 years of a long follow-up, and the apolipoprotein-E (APOE) genotype was assessed. Regions of interest were defined in the bilateral cortex on coregistered MRI. Quantitative analysis of [11C]-PIB was performed using the distribution value ratio (DVR). Longitudinal changes in global and regional PIB DVRs were evaluated in the same regions, and the annual rate of change in the PIB DVR was calculated.

Seven (30.4%) of 23 initially amyloid-negative non-demented subjects converted to globally amyloid-positive (global PIB DVR ≥1.40) over a follow-up of 6.5 ± 1.4 years (converter). The global PIB DVR in converters increased from 1.22 ± 0.07 at baseline to 1.63 ± 0.15 (n = 7, *P* < .01) at last follow-up, and an annual increase of global PIB DVR was 0.057 ± 0.019/year (n = 7, *P* < .01). In contrast, the global PIB DVR in the remaining 16 subjects was 1.15 ± 0.07 at baseline and did not change over a follow-up period (stable). The APOE ε4 allele was present in 4 (57.1%) of the 7 converters, differing from 2 (12.5%) of 16 stable subjects (Fisher's exact test, *P* < .05). Three amyloid-negative MCI subjects had an annual increase in global PIB DVR above 0.061/year and became positive at 2.8 ± 0.5 years of follow-up, which was faster than 5.0 ± 2.0 years in 4 CN subjects. The regional PIB DVR that increased early above the regional positivity threshold was most frequently found in the right lateral temporal cortex (71.4%), followed by the left frontal cortex (41.8%).

Our results suggest that the initially amyloid-negative CN and MCI subjects, especially with APOE ε4, can become globally amyloid-positive over a longer time, based on early regional Aβ deposition in the lateral temporal cortex and/or frontal cortex.

## 1 Introduction

The diagnostic criteria for the spectrum of Alzheimer's disease (AD) supported by biomarkers of the underlying pathophysiological process have been proposed with the National Institute on Aging and the Alzheimer's Association (NIA-AA) workgroup.^[1]^ This disease framework with biomarkers is a principal advancement in clinical and pathophysiological progression of AD. Among the biomarkers, amyloid positron emission tomography (PET) imaging is reliable biomarker for evaluating the amyloid beta (Aβ) plaques of AD neuropathological changes.^[2]^ Therefore, amyloid PET can determine whether individuals are in the spectrum of AD.

The predementia phase in the AD spectrum is part of the clinical and pathophysiological processes of AD. Longitudinal studies have recently demonstrated that individuals with mild cognitive impairment (MCI) or cognitively normal (CN) progressed to the early stage of AD spectrum when Aβ deposition was defined by amyloid PET imaging.^[3,4]^ Amyloid PET imaging can identify the status of Aβ deposition in non-demented individuals and predict the progression to AD dementia. In contrast, if Aβ deposition is not identified by amyloid PET imaging in non-demented individuals, it is unknown whether Aβ deposition will increase among cortical regions over time and be detected with repeated amyloid PET. Furthermore, the earlier stage of Aβ deposition in the cortical region, before Aβ deposition is found throughout cortex, remains poorly understood.

We assessed the longitudinal changes in Aβ deposition in cortical regions with carbon 11 [11C]-labeled Pittsburgh compound B (PIB) PET in initially amyloid-negative non-demented subjects with CN and MCI, and evaluated whether amyloid-negative subjects become globally amyloid-positive and progress into an early stage of AD spectrum over a long time. In addition, we sought to clarify the earlier Aβ deposition in cortical regions before the appearance of widespread cortical Aβ deposition.

## 2 Materials and methods

### 2.1 Subjects

Non-demented subjects, aged 60 to 75 years, were recruited from our memory clinic and through community advertisements. The subjects underwent cognitive tests and amyloid PET imaging using [11C]-PIB. Global cognitive status was evaluated with the Mini-Mental State Examination (MMSE) ^[5]^ and dementia severity was with the Clinical Dementia Rating (CDR) scale and CDR sum of boxes (CDR SB).^[6]^

Twenty-three non-demented subjects who had initially negative [11C]-PIB PET scans were included in the longitudinal study with a long follow-up period of more than 5 years between February 2008 and October 2019. Of these subjects, 16 CN subjects were defined by a CDR score of 0 and a MMSE score of ≥28 and 7 MCI subjects had a CDR score of at least 0.5 in the memory domain and a MMSE score of ≥ 24. All subjects underwent neurological assessment, cognitive test, immediate, and delayed recall of a paragraph from the Wechsler Memory Scale-Revised (WMS-R) Logical Memory II,^[7]^ and [11C]-PIB PET scan at least two or more over the 6.4 ± 1.5 years of follow-up (range: 5.0−9.4 years). AD dementia was defined with a MMSE score of ≤ 23, a CDR score of 0.5 or 1, and a CDR SB score of ≥ 2. The apolipoprotein E (APOE) genotype was assessed at baseline. Participants were excluded if they had any other medical disease, other neurodegenerative conditions, traumatic brain injury, major depression or vascular disease. All participants provided written informed consent. The study was approved by the Ethics Committee of the Mirai Iryo Research Center Inc. (Tokyo, Japan).

### 2.2 PET imaging

PET imaging was conducted using a Siemens ECAT ACCEL scanner with an axial field of view of 155 mm, providing 63 contiguous 2.4 mm slices with a 5.6 mm transaxial and 5.4 mm axial resolution. The subject's head was immobilized with a self-adhering wrap across the forehead to minimize motion during the scan. [11C]-PIB was produced with standard procedures at our PET center, as previously described.^[8]^ [11C]-PIB was injected intravenously with a mean dose of 550.0 ± 10% MBq. Dynamic PET scanning in the 3-dimensional mode was carried out for 60 minutes using a predetermined protocol (31 frames: 4 × 15, 8 × 30, 9 × 60, 2 × 120, and 8 × 300 seconds). Images were reconstructed with an iterative reconstruction algorithm, using the Gaussian filter of 3.5 mm full-width at half-maximum. All subjects underwent volumetric T1-weighted magnetic resonance imaging (MRI) (1.5 Tesla Toshiba scanner) for screening and subsequent coregistration with the PET images.

### 2.3 Image analysis

A region of interest (ROI) analysis was performed on MRI-based correction of PET data using the PMOD software package (PMOD Technologies Ltd., Adliswil, Switzerland). The ROIs were manually drawn on the coregistered MR image, including the bilateral cortical regions: medial temporal cortex, lateral temporal cortex, frontal cortex, parietal cortex, occipital cortex, sensory motor cortex, precuneus cortex, anterior cingulate gyrus, posterior cingulate gyrus, and cerebellar cortex. The same ROIs were applied to both baseline and follow-up images. The retention of [11C]-PIB was determined by the distribution volume ratio (DVR) with Logan graphical analysis for 35 to 60 minutes with cerebellar gray matter as the reference.^[9]^ The regional PIB DVR in each cortical region and the global PIB DVR for the mean of the regional PIB DVR over 18 cortical regions were defined. PIB PET DVR images were created for visual inspection with a rainbow color scale.

### 2.4 Positivity threshold

All subjects were classified as amyloid-positive or amyloid-negative in our clinical setting. The threshold of positivity was defined at 2 standard deviations (SD) above the mean PIB DVR in 34 older healthy control subjects (aged ≥ 56 years), as previously described.^[10]^ The positivity threshold of global PIB DVR was 1.40 while that of regional PIB DVR was different in each cortical region (range: 1.36−1.52).

### 2.5 Data management

The subjects underwent [11C]-PIB PET imaging and clinical assessments at each time point during a follow-up period of more than 5 years. The annual rate of change in PIB DVR was calculated for each subject at follow-up using the following equation: annual rate of change = [(DVR at follow-up − DVR at baseline)/follow-up period (years)].

### 2.6 Statistical analysis

Clinical group differences were evaluated with the Student *t* tests and the changes between baseline and follow-up data were with the paired *t* tests. Multiple comparisons of the differences in cortical regions were performed using the Bonferroni post hoc test. The correlation between PIB DVRs, baseline age, MMSE scores, and WMS-R recall scores were assessed with Pearson's correlation coefficient. Categorical variables were examined with the Fisher's exact test. All analyses were used with Statcel 3 software (OMS Inc., Tokyo, Japan). The results were considered significant at *P* < .05. Continuous variables are presented as the mean ± standard deviation and categorical data are as number (percentage).

## 3 Results

### 3.1 Clinical data and cognitive function

Seven (30.4%) of all 23 initially amyloid-negative non-demented subjects crossed the positivity threshold and converted to globally amyloid-positive over a follow-up period of 7.3 ± 1.2 years (referred to as converter). Four (25%) of 16 amyloid-negative CN subjects and 3 (42.8%) of the 7 MCI subjects were converters. In contrast, the remaining 16 amyloid-negative subjects remained negative over a follow-up of 6.2 ± 1.4 years (referred to as stable).

The demographic characteristics of the converters and stable subjects at baseline and last follow-up are summarized in Table 1. There were no significant differences in age, education level, MMSE, CDR, CDR SB, and WMS-R recall scores at baseline between converters and stable subjects. The proportion of females was significantly greater in converters (85.7%) than in stable subjects (37.5%). The APOE ε4 allele was present in 4 (57.1%) of the 7 converters, being significantly different from 2 (12.5%) of 16 stable subjects (Fisher's exact test, *P* = .045). At last follow-up, the MMSE, CDR, CDR SB, and WMS-R recall scores in converters and stable subjects did not significantly differ from those at baseline.

**Table 1:** Demographic characteristics of initially amyloid-negative subjects at baseline and last follow-up.

	All	Converter	Stable
n	23	7	16
Female	12 (52.1%)	6 (85.7%)^∗^	6 (37.5%)
Age, yr	67.9 ± 5.0	69.2 ± 3.2	67.3 ± 5.6
Education, yr	13.3 ± 2.5	12.8 ± 3.1	13.6 ± 2.2
APOE ε4 carriers	6 (26.0%)	4 (57.1%)^∗^	2 (12.5%)
at baseline			
MMSE	28.2 ± 2.3	27.5 ± 2.7	28.5 ± 2.1
CDR	0.15 ± 0.24	0.21 ± 0.27	0.13 ± 0.22
CDR SB	0.22 ± 0.39	0.43 ± 0.61	0.13 ± 0.22
Immediate rec	12.2 ± 4.9	10.7 ± 4.6	12.9 ± 5.1
Delayed rec	9.0 ± 4.7	7.4 ± 5.0	9.8 ± 4.6
at last follow-up			
MMSE	27.3 ± 3.7 (0.135)	27.8 ± 3.9 (0.407)	27.9 ± 2.1 (0.177)
CDR	0.33 ± 0.47 (0.052)	0.57 ± 0.53 (0.078)	0.19 ± 0.25 (0.163)
CDR SB	0.96 ± 2.53 (0.135)	1.57 ± 2.30 (0.151)	0.31 ± 0.44 (0.082)
Immediate rec	12.0 ± 5.7 (0.835)	9.4 ± 3.8 (0.467)	13.2 ± 5.1 (0.748)
Delayed rec	9.6 ± 6.8 (0.584)	6.5 ± 7.3 (0.701)	11.0 ± 6.3 (0.315)
Follow-up, yr	6.5 ± 1.4	7.3 ± 1.2	6.2 ± 1.4

APOE = apolipoprotein E, CDR = Clinical Dementia Rating, CDR SB = CDR sum of boxes, MMSE = Mini-Mental State Examination, n = number of subjects, rec = WMS-R recall scores, Data are presented as means ± SD.

∗ Fisher's exact test, *P* < .05, *P* value of paired *t* test between baseline and follow-up data is given in parenthesis.

Four CN converters had a mean MMSE score of 29.5 ± 0.5 and a CDR score of 0 at baseline, of whom one progressed to MCI at 5.4 years. In contrast, 3 MCI converters had a mean MMSE score of 25.3 ± 1.5 and a CDR score of 0.5 at baseline, of whom one progressed to AD dementia at 3.1 years.

### 3.2 Global Aβ deposition

**Figure 1:**
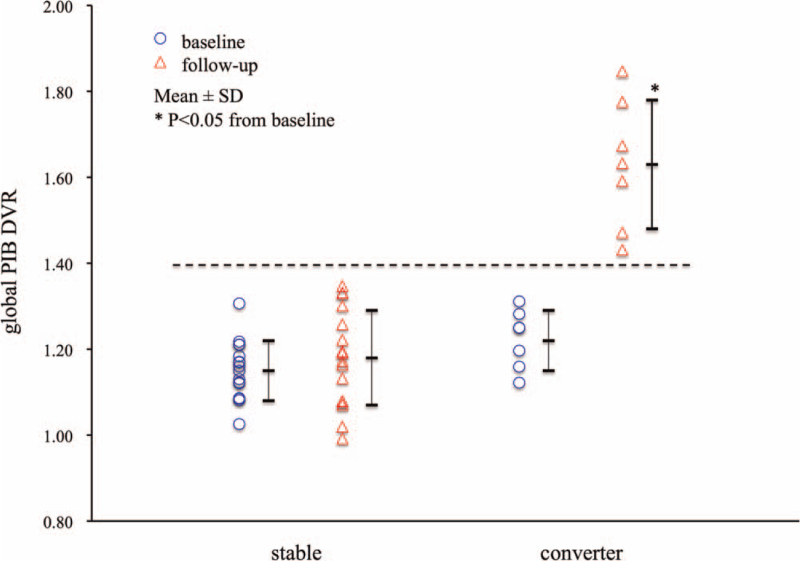
Individual and mean global PIB DVR at baseline and last follow-up in stable subjects and converters. Global PIB DVR in all subjects is below the positivity threshold (dotted line) at baseline, while that in all converters increases at follow-up and is above the threshold.

**Figure 2:**
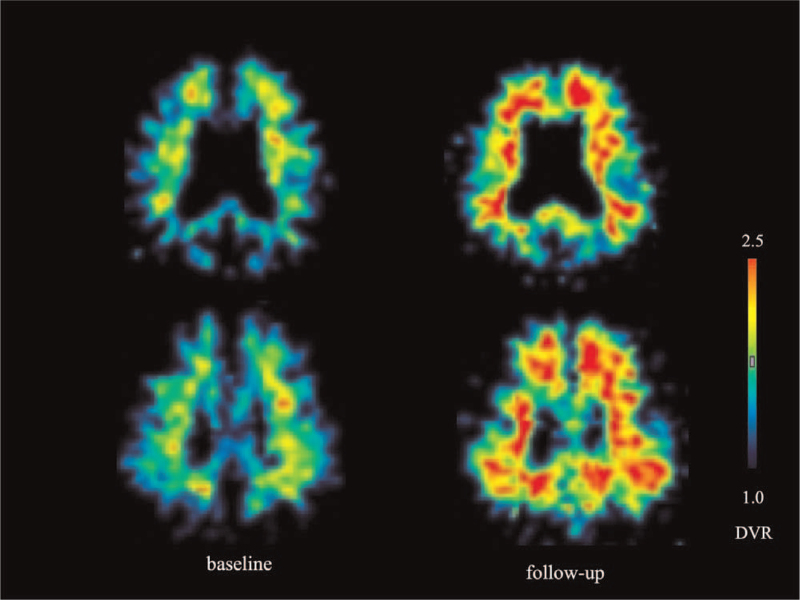
Representative axial [11C]-PIB PET DVR images at baseline and follow-up in the MCI converter. The PIB DVR image at 7.4 years of follow-up shows extensive PIB retention in cortical regions, while the image at baseline shows nonspecific PIB retention in white matter.

The global PIB DVR at baseline and last follow-up in the converters and stable subjects is shown in Figure 1. All initially amyloid-negative non-demented subjects did not have PIB retention in any cortical region at baseline and had global PIB DVR below the positivity threshold. The global PIB DVR at baseline was 1.22 ± 0.07 (n = 7) in converters and 1.15 ± 0.07 (n = 16) in stable subjects. The global PIB DVR at baseline was 1.22 ± 0.09 (n = 6, *P* = .115) in APOE ε4 carriers, being not significantly different from 1.16 ± 0.06 (n = 17) in non-carriers. All converters had a marked PIB retention in the cortical regions at last follow-up, and the representative PIB PET DVR images in the converter at baseline and last follow-up are shown in Figure 2. The global PIB DVR in converters significantly increased to 1.63 ± 0.15 (n = 7, *P* = .0007) at last follow-up. In contrast, in the stable subjects, the global PIB DVR was 1.18 ± 0.11 (n = 16, *P* = .293) at last follow-up, which was not significantly different from that at baseline. Of the 7 converters, the global PIB DVR at baseline in the 4 CN converters was 1.24 ± 0.11, while that in the 3 MCI converters was 1.20 ± 0.08. At last follow-up, global PIB DVR in CN and MCI converters increased to 1.61 ± 0.19 and 1.67 ± 0.10, respectively. There was no significant difference in global PIB DVR at baseline and follow-up between the CN and MCI converters.

**Figure 3:**
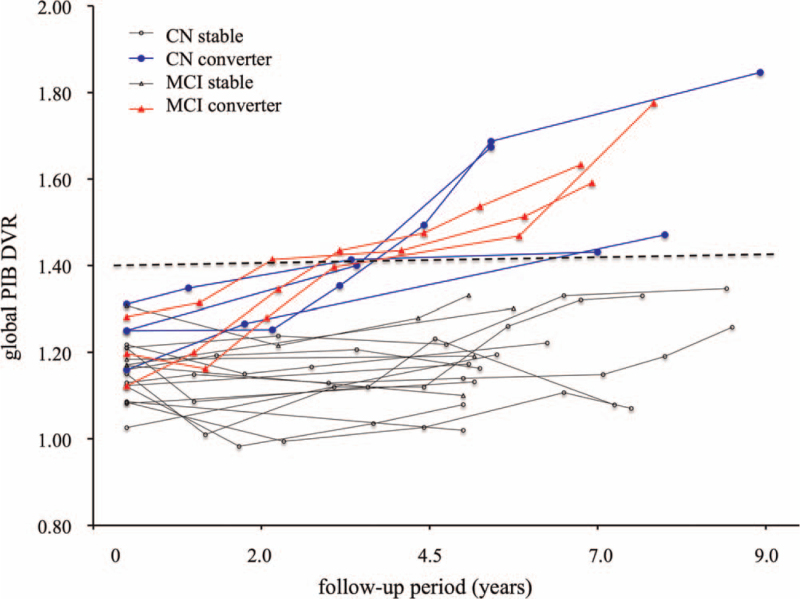
Individual global PIB DVR at various time points over a follow-up period in converters and stable subjects with CN and MCI. The global PIB DVR in all converters continues to increase crosses the positivity threshold (dotted line), while that in stable subjects does not.

Individual global PIB DVRs at various time points of follow-up in all initially amyloid-negative non-demented subjects are shown in Figure 3. The global PIB DVR in all converters continued to increase over the follow-up period, while none of the stable subjects crossed the positivity threshold of the global PIB DVR. The earliest time when global PIB DVR was above the positivity threshold was 2.1 years in the MCI converter, while the slowest time was 8.0 years in the CN converter. The mean follow-up period until global PIB DVR was above the threshold was 2.8 ± 0.5 years in MCI converters, which was earlier than 5.0 ± 2.0 years in CN converters.

### 3.3 Change in global Aβ deposition

The mean annual rate of change in global PIB DVR was 0.057 ± 0.019/year (n = 7, *P* = 1.37E-06) in converters, which was significantly larger than 0.004 ± 0.013/year (n = 16) in stable subjects. The annual increase of global PIB DVR in APOE ε4 carriers of converters was 0.065 ± 0.015/year (n = 4), which was larger than that in non-carriers (0.047 ± 0.022/year, n = 3). An annual increase of global PIB DVR in individual converters did not correlate with baseline global PIB DVR (*r* = −0.62, n = 7, *P* = .13) or baseline age (*r* = 0.16, n = 7, *P* = .72). In addition, the annual change of MMSE scores in individual converters was not correlated with annual changes of global PIB DVR (*r* = −0.30, n = 7, *P* = .50).

**Figure 4:**
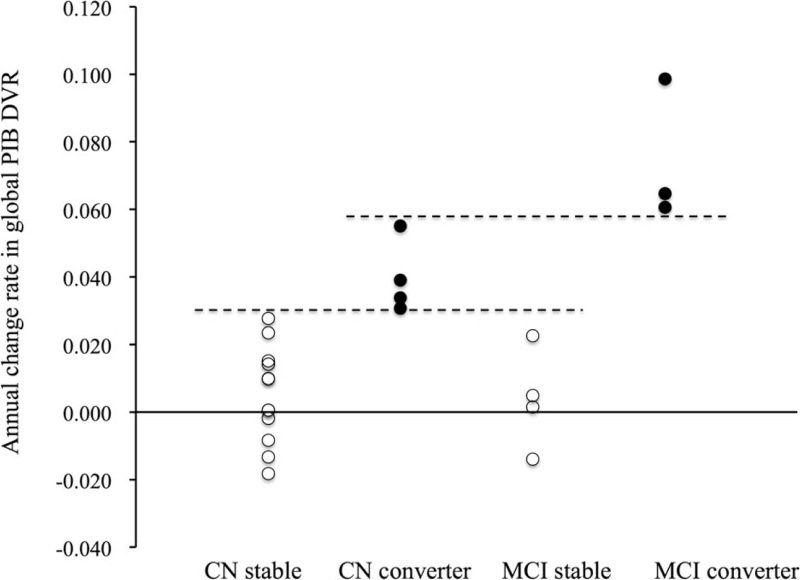
Individual change rate of global PIB DVR until global PIB DVR increases above positivity threshold in stable subjects and converters with CN and MCI. The annual increase of global PIB DVR is above 0.031/year (lower line) in all converters, of which it is above 0.061/year (upper line) in MCI converters and larger than in CN converters.

The annual change of global PIB DVR until global PIB DVR increased above the positivity threshold in converters and stable subjects with CN and MCI is shown in Figure 4. The annual increase of global PIB DVR was above 0.031/year in all converters, while it was below 0.027/year in all of stable subjects. All MCI converters had an annual increase in global PIB DVR above 0.061/year, which was larger than CN converters.

### 3.4 Regional Aβ deposition

The regional PIB DVR in all cortical regions at baseline was below the positivity threshold in each cortical region in converters and stable subjects. At last follow-up, the regional PIB DVR in converters increased above the positivity threshold in each cortical region, except for the medial temporal, occipital, and sensory motor cortices. In contrast, the regional PIB DVR in stable subjects did not increase in any cortical region.

Among all cortical regions, 4 cortical regions of the lateral temporal, frontal, parietal, and precuneus cortices were evaluated as AD signature regions. Regional PIB DVR at baseline and annual rate of change in regional PIB DVR in converters and stable subjects are summarized in Table 2. The regional PIB DVR at baseline did not significantly differ in cortical regions between converters and stable subjects. The annual increase in regional PIB DVR in each cortical region was significantly large in converters compared with that in stable subjects. In particular, the annual increase in regional PIB DVR was greatly found in the right precuneus and left frontal cortex of converters, but there was no significant difference in the annual increase of regional PIB DVR among these cortical regions.

**Table 2:** Regional PIB DVR at baseline and the annual rate of change in regional PIB DVR in converters and stable subjects.

	Baseline		Annual rate of change	
	converter	stable	converter	stable
n	7	16	7	16
R.LTC	1.24 ± 0.12	1.14 ± 0.08	0.054 ± 0.038^∗^	0.008 ± 0.024
L.LTC	1.21 ± 0.10	1.04 ± 0.09	0.060 ± 0.031^∗^	0.013 ± 0.021
R.FC	1.29 ± 0.04	1.17 ± 0.10	0.070 ± 0.047^∗^	−0.001 ± 0.026
L.FC	1.29 ± 0.15	1.13 ± 0.11	0.076 ± 0.051^∗^	0.001 ± 0.028
R.PC	1.15 ± 0.16	1.11 ± 0.12	0.063 ± 0.037^∗^	0.005 ± 0.028
L.PC	1.21 ± 0.10	1.08 ± 0.11	0.064 ± 0.036^∗^	0.015 ± 0.024
R.Pre	1.24 ± 0.13	1.19 ± 0.12	0.079 ± 0.033^∗^	0.002 ± 0.025
L.Pre	1.21 ± 0.13	1.20 ± 0.12	0.076 ± 0.038^∗^	0.001 ± 0.024

Data are presented as mean ± SD. FC = frontal cortex, L = left, LTC = lateral temporal cortex, n = number of subjects, PC = parietal cortex, Pre = precuneus, R = right.

∗ Statistically significant difference from stable by Student *t* test (*P* < .05).

**Figure 5:**
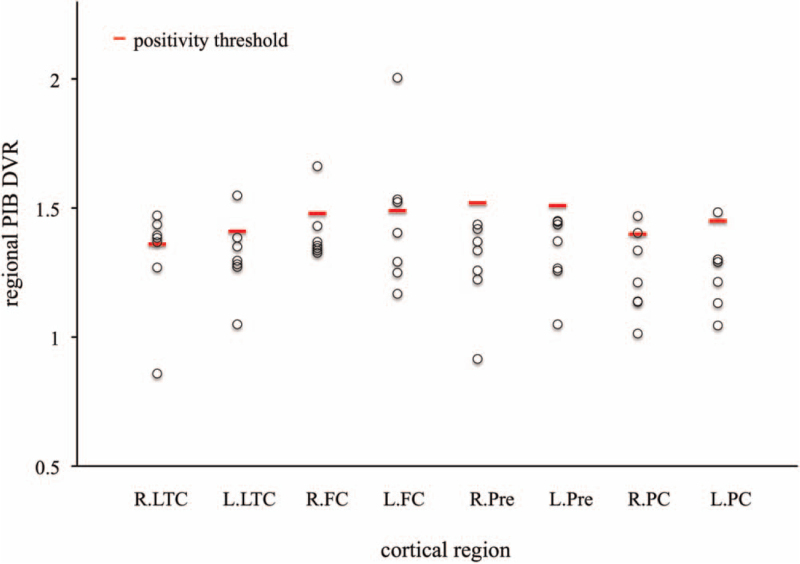
Individual regional PIB DVR in each cortical region in the converters before global PIB DVR increases above the positivity threshold. Regional PIB DVR that is the earliest above regional positivity threshold is most frequently found in right lateral temporal cortex. FC = frontal cortex, L = left, LTC = lateral temporal cortex, PC = parietal cortex, Pre = precuneus, R = right.

To identify early Aβ deposition regions, before global PIB DVR increased above the positivity threshold, the regional PIB DVR was analyzed among four cortical regions in seven converters, and the results are shown in Figure 5. The earliest region that regional PIB DVR was above the threshold of amyloid positivity in each region was the right lateral temporal cortex in 5 (71.4%) converters at 1.8 ± 0.8 years of follow-up, followed by the left frontal cortex in 3 (42.8%) converters at 2.8 ± 1.6 years.

## 4 Discussion

We demonstrated that 7 (30.4%) of 23 initially amyloid-negative non-demented subjects became amyloid-positive over a follow-up period of 7.3 ± 1.2 years. A previous [11C]-PIB PET study revealed that 14 (11.1%) of 126 initially amyloid-negative CN participants crossed the low threshold for PIB positivity after 4 years of follow-up.^[11]^ It has also been reported, in the Alzheimer's Disease Neuroimaging initiative (ADNI) cohort, that 13 (9.2%) of 142 florbetapir negative CN individuals converted to florbetapir positive status over 3.9 ± 1.4 years.^[12]^ In this study, 4 (25%) of 16 amyloid-negative CN subjects and 3 (42.8%) of 7 amyloid-negative MCI subjects converted to amyloid-positive. These findings suggest that even if CN subjects, in addition to MCI subjects, are amyloid-negative at baseline with amyloid PET, they can become amyloid-positive over a longer time.

Furthermore, in the present study, 4 CN converters were defined as preclinical AD by NIA-AA criteria, of whom 1 progressed to MCI due to AD at 5.4 years of follow-up, while 3 MCI converters were defined as MCI due to AD, of whom 1 progressed to AD at 3.1 years. We have recently reported that 12 (54.5%) of 22 subjects with preclinical AD progressed to MCI due to AD within 7 years, of whom two progressed to AD.^[4]^ Also, we reported that 28 (71.7%) of 39 patients with MCI due to AD progressed to AD within 8 years.^[3]^ Therefore, if amyloid-negative CN or MCI subjects become globally amyloid-positive, they will progress to an early stage of AD spectrum over time.

Genetically, the ε4 allele of the APOE gene is known to be stronger risk factor for AD. The frequency of the APOE ε4 allele is markedly increased to more than 40% in patients with AD, although the APOE ε4 allele has a worldwide frequency of 13.7%.^[13]^ Our previous study reported that APOE ε4 carriers were 53.8% of 26 amyloid-positive CN, MCI and AD patients, who had a larger annual increase in Aβ deposition.^[14]^ In the present study, in contrast, an APOE ε4 allele was present in 57.1% of amyloid-negative CN and MCI subjects who converted to amyloid-positive over time. The annual increase of global PIB DVR in APOE ε4 carriers was larger than that in non-carriers, although the PIB DVR at baseline in APOE ε4 carriers did not differ from that in non-carriers. These findings suggest that the APOE ε4 allele might be involved in initiating Aβ deposition, in addition to accelerating Aβ aggregation in the brain.

The present study found that amyloid-negative non-demented subjects who converted to amyloid-positive had a significantly larger annual increase of global PIB DVR. In particular, until the global PIB DVR was above the positivity threshold, the annual increase in global PIB DVR in amyloid-negative MCI subjects was larger than that in amyloid-negative CN subjects, and its period was shorter in MCI subjects than in CN subjects. These findings indicate that amyloid-negative MCI subjects become amyloid-positive faster based on a larger annual increase in global PIB DVR.

We demonstrated that the annual increase of regional PIB DVR was larger in the right precuneus and left frontal cortex of converters but did not significantly differ among cortical regions. Furthermore, in this study, before the global PIB DVR was above the positivity threshold, the regional PIB DVR that increased early above the regional threshold of amyloid positivity was most frequently found in the right lateral temporal cortex (71.4%), followed by the left frontal cortex (41.8%). Another PIB PET study with spatial distribution reported that the percentage of regionally elevated PIB uptake in cortical regions for old normal control subjects with global PIB DVR (below the threshold for positivity) was 29% in the left parietal cortex, 20% in the left prefrontal cortex, and 20% in the right lateral temporal cortex.^[15]^ These findings indicate that the lateral temporal and/or frontal cortices might be the initial cortical regions that the Aβ deposition increases regionally. In contrast, the study with florbetapir PET-based amyloid staging in the ADNI cohort has reported that early amyloid deposition was found in the basal part of the temporal lobe, the anterior cingulate gyrus, and parietal operculum.^[16]^ These discrepancies are likely to be the different analytic approaches with radiotracer-related differences. We suggest that amyloid-negative subjects would become globally amyloid-positive over a longer time, if amyloid-negative non-demented subjects are longitudinally evaluated with amyloid PET and Aβ deposition is detected regionally in some cortical regions.

Brain Aβ deposition has been proposed to initiate a downstream mechanism that affects symptom progression. Disease-modifying therapy for AD has been developed for clinical use and has moved to target patients in the earlier stage of the AD continuum. Recently, the PRIME phase 1b study of the anti-Aβ antibody (BIB037) has shown, in the prodromal or mild AD patients with positive Aβ PET scans, that the anti-Aβ antibody significantly reduced beta-amyloid plaques in cortical regions over 1 year.^[17]^ The present study found that amyloid-negative non-demented subjects became globally amyloid-positive over time, based on early Aβ deposition in the lateral temporal cortex and/or frontal cortex. Therefore, if the monoclonal antibody treatment with Aβ plaque removal can be given in the amyloid-negative individuals who early regional Aβ deposition is detected in some cortical regions by amyloid PET, these individuals will not have the Aβ deposition spread in the association neocortex.

Certain limitations of our study should be considered. We have successfully assessed the longitudinal changes of Aβ deposition in cortical regions with [11C]-PIB PET over a long time in initially amyloid-negative non-demented subjects. The number of subjects included in this study was comparatively small, particularly for subjects who converted to globally amyloid-positive. Further studies with larger populations are necessary to confirm these findings. The quantitative assessment was conducted by PIB DVR normalized to cerebellar gray matter as a reference. Also, the amyloid-positive threshold of regional and global PIB DVR was determined in our clinical setting. The threshold for positivity was relatively high because it was driven by a group of older healthy control subjects (aged ≥56 years) to adjust for the elderly subjects enrolled in this study.

## 5 Conclusion

The initially amyloid-negative non-demented subjects, especially with APOE ε4, could become globally amyloid-positive over a longer time and progress to an early stage of AD spectrum. The MCI subjects become amyloid-positive faster than CN subjects based on a larger annual increase in Aβ deposition. When Aβ deposition is regionally located in the lateral temporal and/or frontal cortex, it will be widely distributed in the association neocortex over time. Therefore, even baseline-negative CN and MCI subjects are needed to repeatedly have the amyloid PET over a long time for an early intervention before downstream effects of Aβ deposition.

## Author contributions

DW provided the data acquisition. SH conducted the data analysis and interpretation, and wrote the paper. SH reviewed and edited the manuscript.

**Data curation:** Daichi Wakebe.

**Investigation:** Shizuo Hatashita.

**Validation:** Shizuo Hatashita.

**Writing – original draft:** Shizuo Hatashita.

**Writing – review & editing:** Shizuo Hatashita.
